# P-1726. Antibiotics Prescribing Practices for Skin and Soft Tissue Infections in an Urban Urgent Care Setting

**DOI:** 10.1093/ofid/ofae631.1890

**Published:** 2025-01-29

**Authors:** Chuman Xie, Amanda Devlin, Sanjana Koshy, Erick Eiting, Jason Hedvat

**Affiliations:** Mount Sinai Beth Israel, New York, New York; Mount Sinai Beth Israel, New York, New York; Mount Sinai Beth Israel, New York, New York; Mount Sinai Beth Israel, New York, New York; Mount Sinai Beth Israel, New York, New York

## Abstract

**Background:**

Skin and soft tissue infections (SSTIs) are amongst the most common bacterial infections in the ambulatory care setting. Initial antibiotic treatment should consider anticipated pathogens, severity, patient-specific considerations, and local antibiogram data. This study evaluated the alignment of antibiotic prescription practices for SSTIs with IDSA guidelines to identify opportunities to improve antibiotic prescribing at a single urgent care (UC) center.

**Methods:**

This was a retrospective study conducted at the UC in an urban academic health system of adult patients with SSTI diagnosis receiving at least one antibiotic prescription from July 1, 2022, to August 31, 2023. The primary outcome was the proportion of patients with antibiotic prescriptions deviated from IDSA guidelines, considering individual patient factors and local antibiogram data. Secondary outcomes included evaluating the use of clindamycin when better alternatives were available, combination therapy when monotherapy would have been sufficient, deviations from guideline-recommended dosage regimen, and bug-drug mismatch rates. Descriptive statistics were used for outcome analysis.

**Results:**

141 patients were included in the final analysis; 80 patients with purulent SSTIs and 61 patients with nonpurulent SSTIs. Incision and drainage occurred in 70% of patients with purulent SSTIs, but only 11% of patients had cultures obtained. The most common antibiotics prescribed were cephalexin (41%), trimethoprim-sulfamethoxazole (TMP/SMX) (40%), and doxycycline (20%) for purulent SSTIs, and cephalexin (56%) and TMP/SMX (25%) for nonpurulent SSTIs. 50% of patients received antibiotic prescriptions not aligned with guidelines, accounting for individual patient factors and local antibiogram data. 8% of patients were prescribed clindamycin when a better alternative existed. Combination antibiotic therapy was used in 12% of patients where monotherapy would suffice. 10% of patients with purulent SSTIs and 15% with nonpurulent SSTIs received antibiotics without a guideline-recommended dosage regimen.

**Conclusion:**

This study demonstrated that there is room for improvement to align with IDSA guidelines at the UC. Additional provider education on appropriate antibiotic prescribing for SSTIs is warranted.

**Disclosures:**

**All Authors**: No reported disclosures

